# Supplementary material for: Thermoelectric signature of the chiral anomaly in Cd3As2
Source: Nat Commun. 2016 Oct 10;7:13013. doi: 10.1038/ncomms13013 (PMC5062373; doi:10.1038/ncomms13013)
Supplement: Supplementary Information — Supplementary Figures 1-6, Supplementary Notes 1-6 and Supplementary References [file ncomms13013-s1.pdf]

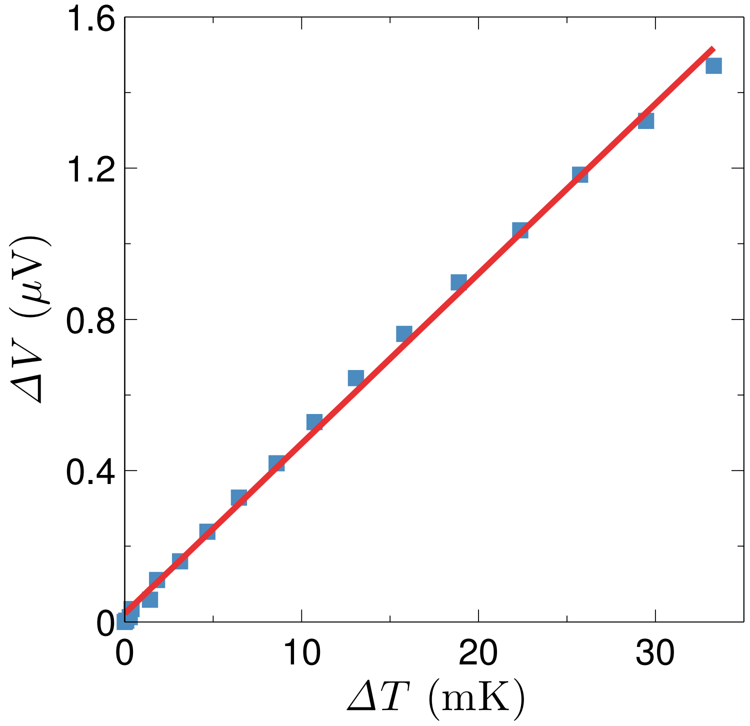

Supplementary Figure 1: Thermoelectric signal. The thermopower voltage ΔV as a function of the temperature difference ΔT measured at 50 K. The red line is a linear fit.

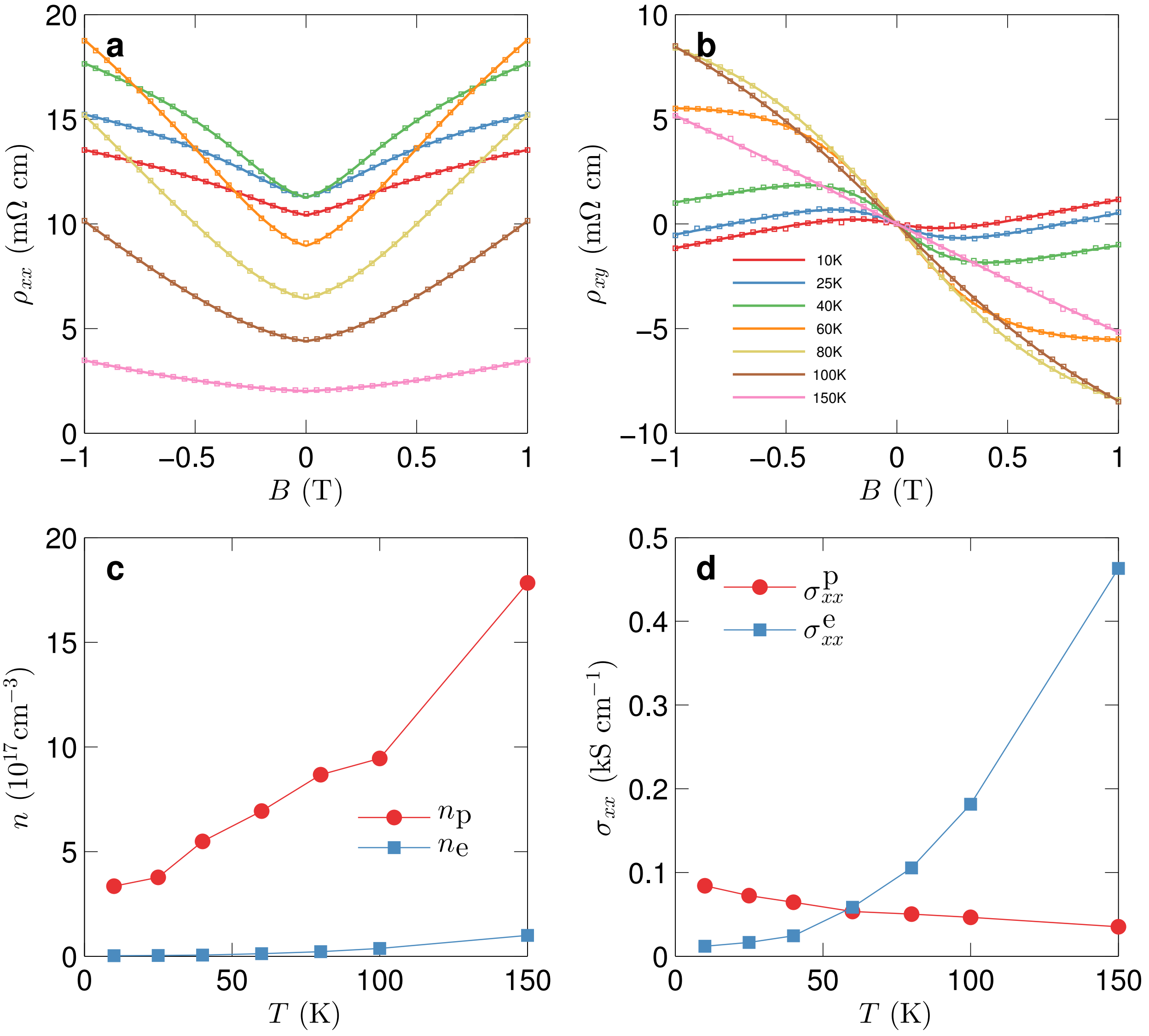

Supplementary Figure 2: Two-band transport at different temperatures. Longitudinal (a) and transverse (b) resistivity as a function of the magnetic field at different temperatures. Open symbols are experimental data and solid lines are the best fits to Supplementary Equation 2. (c) Temperature dependence of the carrier density of holes (n_{p}) and electrons (n_{e}). (d) Temperature dependence of the conductivity of holes (σ_{xx}^{p}) and electrons (σ_{xx}^{e})

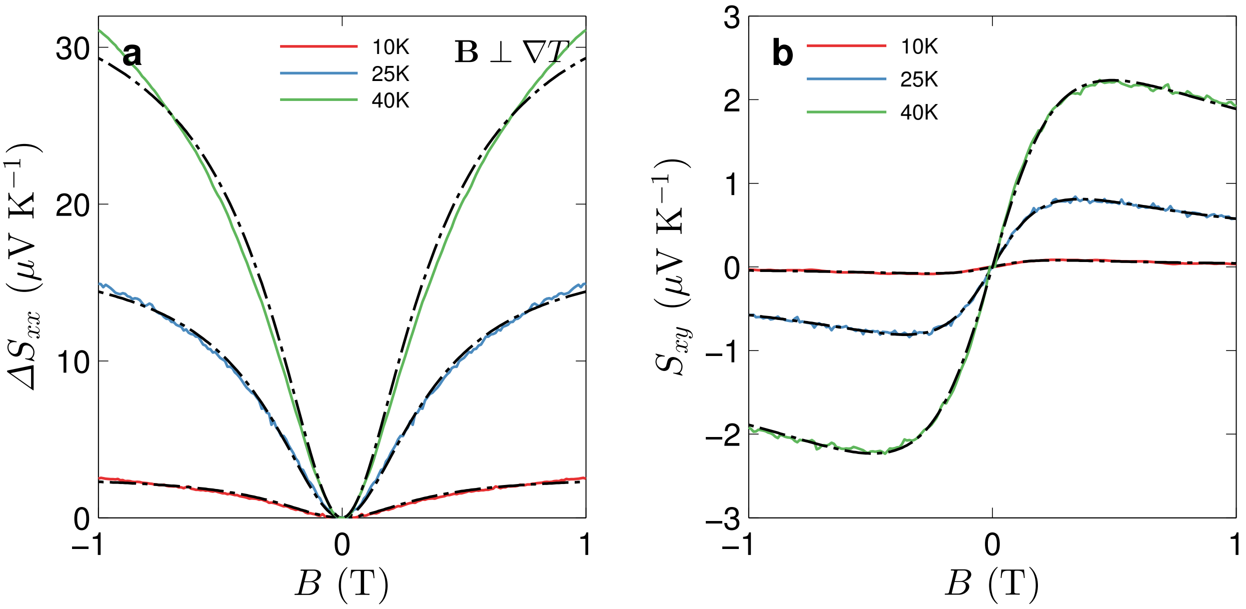

Supplementary Figure 3: Fitting the field dependence of the thermoelectric responses. (a)ΔS_{xx}. (b) Nernst effect S_{xy}. B is perpendicular to ∇T. The dash-dotted lines are fits to Supplementary Equation 3, with a temperature dependent τ.

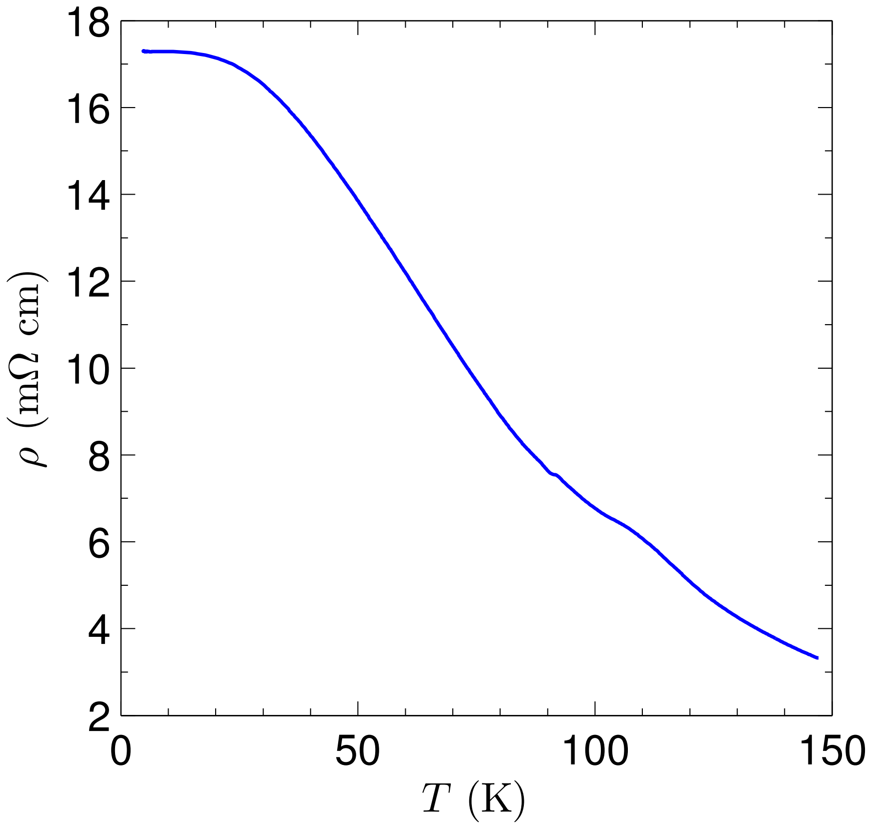

Supplementary Figure 4: Temperature dependence of the resistivity for Sample S3.

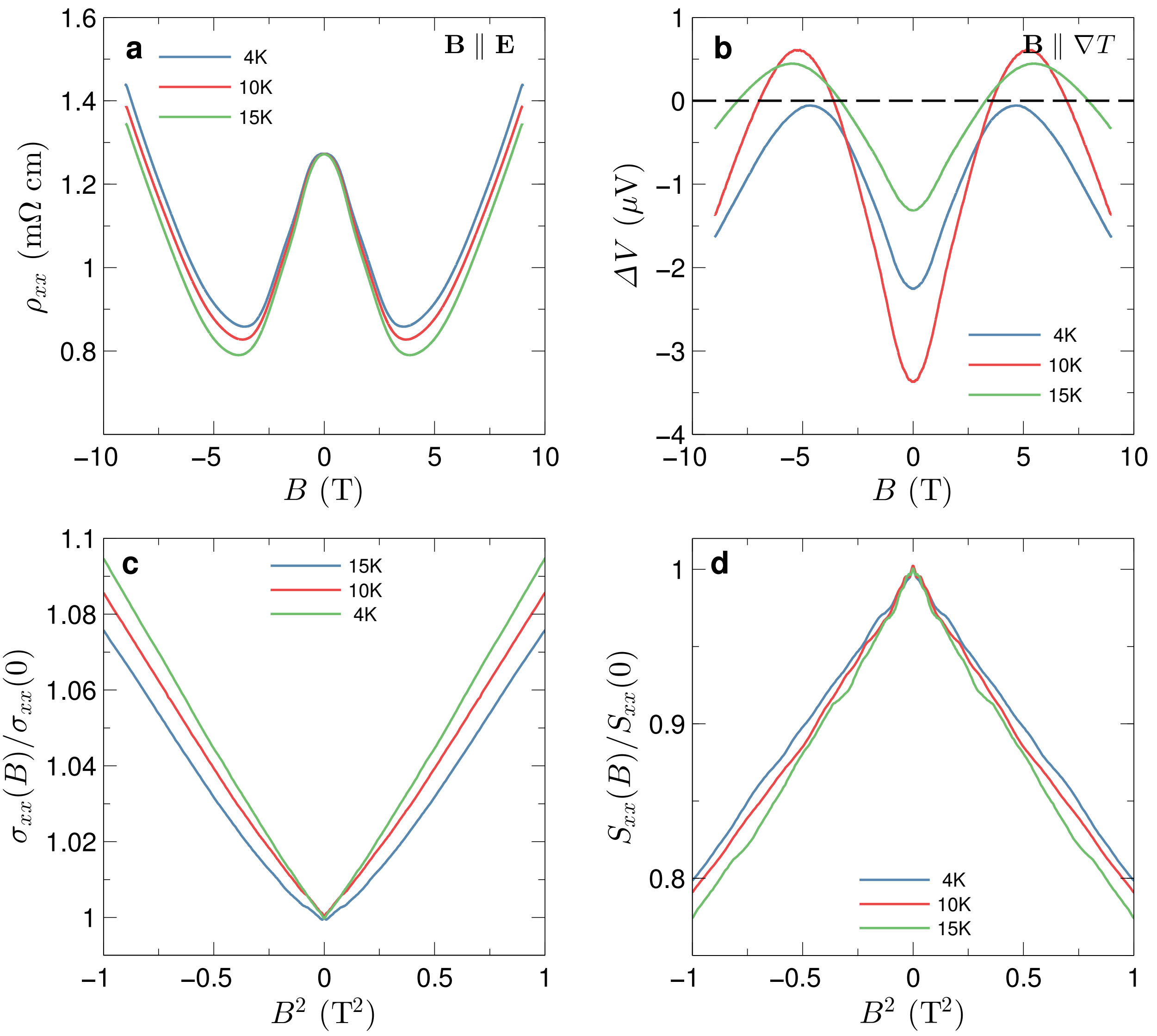

Supplementary Figure 5: Chiral anomaly induced transport for sample S3 under $\mathbf{B} \parallel \mathbf{E}$. Magnetoresistance (a) and thermoelectric signal (b). Since below 10 K, the two on-chip thermometers fail due to a zero temperature coefficient of resistance, we could not get the temperature difference, hence the absolute value of S_{xx}. We only plot the signal $\varDelta V$ in (b). Normalized magnetoconductivity (c) and thermopower (d) as a function of B^2 in low fields.

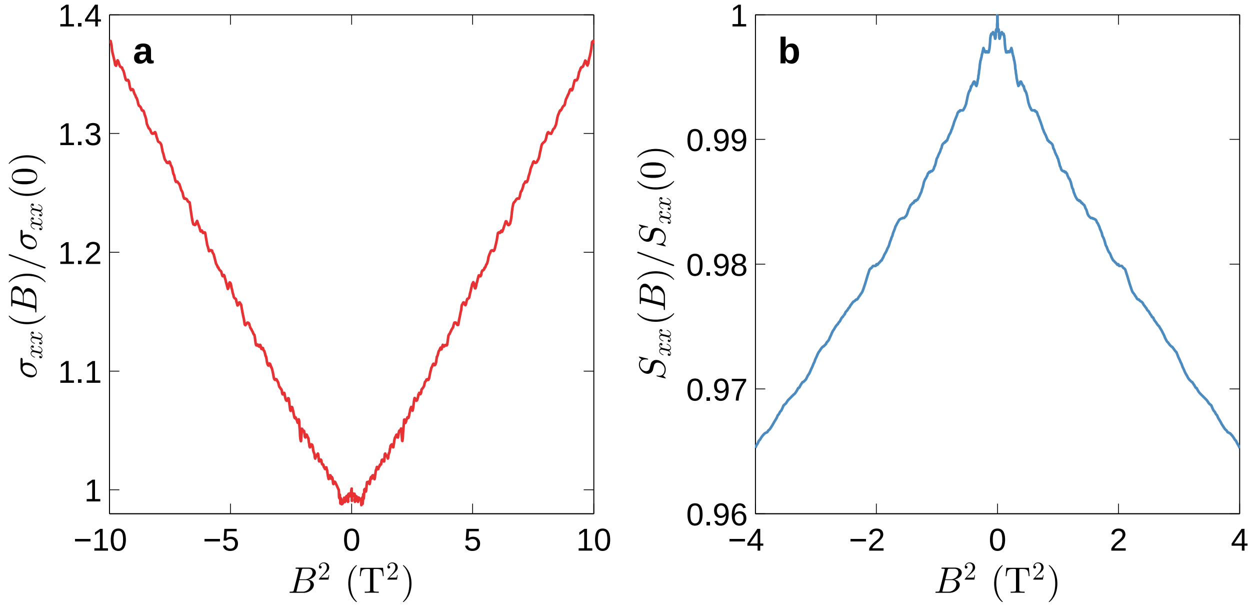

Supplementary Figure 6: Magneto-conductivity and thermopower as a function of B^2 at 150 K.

Supplementary Note 1. Thermoelectric measurements

To measure the temperature difference and the thermoelectric voltage with high signal to noise ratio, a 2ω method was used. An *ac* current at a low frequency of $\omega/2\pi = 1.77$ Hz is injected through the heater, which produces a temperature oscillation at 2ω with a phase shift of $-\pi/2$. The resultant oscillation of the thermometer resistance is measured by passing a *dc* current and detecting the 2ω voltage. Similarly, the thermoelectric voltage is detected at 2ω. Supplementary Fig. 1 shows the measured thermopower voltage as a function of the temperature dependence at 50 K. The linearity of the voltage to ΔT indicates the thermoelectric origin of the signal. Below 10 K, the thermometers stop working because the resistance saturates. So, the thermoelectric measurements were carried out above 10 K.

Supplementary Note 2. Two-band analysis

The carrier density of our Cd_3As_2 micro-plates is relatively low, so the Fermi level is close to the Dirac point. At low temperatures, holes are the dominant carriers. As temperature increases, more and more electrons are thermally excited. Since the mobility of electrons are much higher than that of holes, they eventually dominate the transport. The two-band transport is evident in the temperature dependence of the resistivity and thermopower. It is also reflected in the magnetic field dependence of the thermopower, as we have discussed in the main text. Here, we present the longitudinal (ρ_{xx}) and transverse resistivity (ρ_{xy}) as a function of the magnetic field. By fitting to a two-band model, the switching of the dominant carrier is unambiguously shown.

In a Drude model under a magnetic field B, the resistivity can be written as a tensor

$$\begin{cases} \rho_{xx} = (1 + k_{\mathrm{MR}}B)/ne\mu \\ \rho_{xy} = B/ne \end{cases} . \qquad \text{(Supplementary Equation 1)}$$

Here, the term $k_{\mathrm{MR}}B$ phenomenologically accounts for the linear magnetoresistance widely observed in Cd_3As_2. When there are both an electron band and a hole band, the tensor of the total resistivity is

$$\frac{1}{\boldsymbol{\rho}} = \frac{1}{\boldsymbol{\rho}_{\mathrm{e}}} + \frac{1}{\boldsymbol{\rho}_{\mathrm{p}}}. \qquad \text{(Supplementary Equation 2)}$$

We fit both ρ_{xx} and ρ_{xy} simultaneously, shown in Supplementary Fig. 2a and b. The carrier density and conductivity for electrons and holes as a function of temperature are depicted in Supplementary Fig. 2c and d. Both types of carriers display a significant increase of the density, resulting from thermal activation. However, the increase is different for two, suggesting existence of impurity levels[1]. From the conductivity, it is clear that the dominant carriers switch from holes to electrons as the temperature is raised, supporting our two-band picture.

Supplementary Note 3. Effect of the energy dependence of τ on thermoelectric effects

As the Mott relation indicates, the thermoelectric response is proportional to the derivative of the conductivity with respect to energy ϵ. Therefore, it is strongly affected by the energy dependence of τ. Let us assume a simple form $\tau = C_\tau \epsilon^p$ and take into account the Dirac band dispersion $\epsilon = \hbar k v_{\mathrm{F}}$, then the mobility $\mu = e\tau v_{\mathrm{F}}^2/\epsilon_{\mathrm{F}} = eC_\tau v_{\mathrm{F}}^2 \epsilon_{\mathrm{F}}^{p-1}$. Plugging it into the Supplementary Eqs. 2 and 3 in the main text, we arrive

$$\begin{cases} S_{xx} = \dfrac{\pi^2 k_{\mathrm{B}}^2 T}{3e\epsilon_{\mathrm{F}}} \cdot \left(3 + \dfrac{p-1}{1+\mu^2 B^2}\right) \\ S_{xy} = \dfrac{\pi^2 k_{\mathrm{B}}^2 T}{3e\epsilon_{\mathrm{F}}} \cdot \dfrac{(p-1)\mu B}{1+\mu^2 B^2} \end{cases} \qquad \text{(Supplementary Equation 3)}$$

for a perpendicular magnetic field. A very important feature of this equation is that there is a critical value for p. When $p > 1$, S_{xx} decreases with B. Only when $p < 1$, S_{xx} increases with B, which is observed in our experiment. This feature was also briefly mentioned in Ref. 2. p is usually larger than -1. Therefore, our assumption of $p = 0$ is reasonable. Interestingly, when $p = 1$, S_{xx} is independent of B and S_{xy} is zero. Supplementary Equation 3 is for two-dimension and the critical value of p will be different for systems with different dimensions.

Supplementary Note 4. Effect of the field dependence of τ on thermoelectric effects

In the main text, we analyse the thermoelectric effects under a perpendicular field using a simple Drude model, without considering the field dependence of τ. However, it is known that Cd_3As_2 displays a large linear magnetoresistance. Here, we discuss the effect of the

field dependence. Now, the scattering time is $\tau = \tau_0/(1 + k_{\mathrm{MR}}B)$, as in Supplementary Equation 1. Similarly, the mobility becomes $\mu = \mu_0/(1 + k_{\mathrm{MR}}B)$. Assuming the coefficient k_{MR} is independent of energy, Supplementary Equation 3 still holds with μ being replaced by the magnetic field dependent one. Below 1 T, the magnetoresistance of the sample is not very stong, $k \sim 0.28$. We find that the effect of the field dependence has a marginal effect on our fitting, see Supplementary Fig. 3. Fitting results are similar. The fitted mobility changed by less than 10%.

Supplementary Note 5. Measurements on another sample

Similar chiral anomaly induced transport are also observed in two other samples we have measured. Supplementary Fig. 4 shows the resistivity of sample S3 as a function of temperature. The resistivity saturates at about 20 K, indicating a carrier density even lower than the sample in the main text. Therefore, the analysis based on a one-band model is limited to lower temperatures for S3.

Under a parallel magnetic field, a strong negative magnetoresistance is observed, followed by an increase in high fields. The behaviour is consistent with other samples. As shown in Supplementary Fig. 5c, the magnetoconductivity is proportional to B^2, in agreement with the chiral anomaly induced negative magnetoresistance described by Eq. 4. The magneto-thermopower is also very similar to other samples. We observe a strong decrease of S_{xx} with the field. At $T = 10, 15$ K, it reverses its sign at about 3 T. As we have discussed in the main text, the unusual field dependence can be explained by the chiral anomaly. According to Eq. 7, S_{xx} follows a B^2 dependence in low fields. This is confirmed in Supplementary Fig. 5d, in which S_{xx} is plotted against B^2 and a linear behaviour is observed. The ratio of the coefficients, $C_{\mathrm{MS}}/C_{\mathrm{MR}} \sim 2.4$, in a reasonable agreement with Eq. 7.

Supplementary Note 6. Thermopower at high temperatures

In presence of two bands that are comparable in transport, the thermoelectric effects is complex, which prevents a straightforward analysis. In the main text, we analyze our low temperature data based on a single hole band, as the contribution of electrons are negligible. At high temperatures, electrons will dominate the transport, so a single band analysis should

work again. Show in Supplementary Fig. 6, the conductivity and the thermopower in a parallel field at 150 K are plotted against B^2. Both are linear, in a agreement with Eqs. 5 and 7, supporting the chiral anomaly induced transport. However, the ratio between two coefficients, $C_{\mathrm{MS}}/C_{\mathrm{MR}} \sim 0.2$, significantly less than 2. We are not sure about the origin of the discrepancy. It is possible that thermal smearing of the chiral anomaly and the change of the dominant scattering mechanism play a role. A systematic study of the temperature dependence can provide a clue. To avoid the effect of two-band, such experiments should be carried out in a sample whose transport is dominated by only one band.

Supplementary References

[1] Li, H. *et al.* Negative magnetoresistance in Dirac semimetal Cd_3As_2. *Nat. Commun.* **7**, 10301–10301 (2016).

[2] Liang, T. *et al.* Evidence for massive bulk Dirac fermions in $Pb_{1-x}Sn_xSe$ from Nernst and thermopower experiments. *Nat. Commun.* **4**, 2696 (2013).
